# Comparison of Thermal Stability Between Immersion Bath and Sponge Bath Followed by Kangaroo Mother Care in Stable Preterm in Postnatal Ward

**DOI:** 10.7759/cureus.68230

**Published:** 2024-08-30

**Authors:** Raju Veeraiah, Sharanabasavesh M Mangalgi, Nischal Puttaswamy, Satishkumar K M., Pradeep G C. Maralusidappa, Krithika M Veerabhadraiah

**Affiliations:** 1 Pediatrics and Neonatology, Vijayanagara Bruhat Bengaluru Mahanagara Palike (BBMP) Hospital, Bengaluru, IND; 2 Pediatrics and Neonatology, M S Ramaiah Hospital, Bengaluru, IND

**Keywords:** axillary temperature, kangaroo mother care, hypothermia, thermal stability, sponge bath, immersion bath, preterm neonates

## Abstract

Background: Preterm neonates are at high risk of hypothermia, which can lead to adverse health outcomes. This study aimed to compare the effects of immersion bath followed by Kangaroo Mother Care (KMC) versus sponge bath followed by KMC on the thermal stability of preterm neonates.

Methods: A prospective interventional study was conducted on 76 preterm neonates, with 38 neonates in each group (immersion bath and sponge bath). Axillary temperature was measured before the bath and at various time intervals after the bath (immediately, 15, 30, 45, and 60 minutes).

Results: The mean axillary temperature was significantly higher in the immersion bath group compared to the sponge bath group immediately after the bath (97.6°F vs. 96.3°F, p<0.001), at 15 minutes (97.6°F vs. 96.8°F, p<0.001), 30 minutes (97.9°F vs. 97.3°F, p=0.001), and 45 minutes (98.1°F vs. 97.7°F, p=0.002) post-bath. However, the difference was not statistically significant at 60 minutes post-bath (98.2°F vs. 98.0°F, p=0.091).

Conclusion: Immersion bathing followed by KMC is more effective than sponge bathing followed by KMC in maintaining thermal stability in preterm neonates. Healthcare providers should consider adopting this approach as the preferred bathing method for preterm neonates to promote better health outcomes.

## Introduction

Preterm birth, defined as birth before 37 completed weeks of gestation, is a significant global health concern. According to the World Health Organization (WHO), an estimated 15 million babies are born preterm annually, and this number is rising [1]. Preterm infants are particularly vulnerable to various complications, including hypothermia, which can lead to increased morbidity and mortality [2]. Maintaining thermal stability is crucial for the survival and healthy development of preterm infants.

Kangaroo Mother Care (KMC), a technique that involves skin-to-skin contact between the infant and the mother or caregiver, is an effective method for promoting thermal stability in preterm infants [3]. KMC has been associated with a reduced risk of hypothermia, improved breastfeeding rates, and better growth and development outcomes in preterm infants [4]. The WHO recommends KMC as a standard of care for preterm infants, particularly in low-resource settings [5].

Bathing is an essential aspect of newborn care, but it can also pose a significant challenge to maintaining thermal stability in preterm infants. Preterm infants have a higher body surface area-to-weight ratio, thinner skin, and less subcutaneous fat compared to term infants, making them more susceptible to heat loss during bathing [6]. Therefore, it is crucial to identify bathing methods that minimize heat loss and promote thermal stability in preterm infants.

Two commonly used bathing methods for preterm infants are immersion baths and sponge baths. An immersion bath involves dipping the infant in a tub of warm water, while a sponge bath involves washing the infant with a wet cloth or sponge [7]. Studies have shown that immersion baths may be associated with better thermal stability compared to sponge baths in preterm infants [8,9]. However, there is limited evidence comparing the effects of immersion bath and sponge bath when followed by KMC.

The combination of bathing and KMC may have a synergistic effect on promoting thermal stability in preterm infants. A study by Freitas et al. found that preterm infants who received a tub bath followed by KMC had higher axillary and abdominal temperatures compared to those who received a sponge bath followed by radiant warmer care [10]. However, this study did not compare the effects of immersion bath and sponge bath when both were followed by KMC.

The present study aims to address this gap in knowledge by comparing the effects of immersion bath followed by KMC versus sponge bath followed by KMC on the thermal stability of preterm infants. The primary objective is to compare the axillary temperature of preterm infants at 15, 30, 45, and 60 minutes after immersion bath followed by KMC versus sponge bath followed by KMC. The secondary objectives are to compare the heart rate and oxygen saturation levels of preterm infants between the two groups.

The findings of this study will provide valuable evidence to guide clinical practice and inform the development of evidence-based guidelines for bathing and thermal care of preterm infants. By identifying the optimal combination of bathing methods and thermal care, this study has the potential to improve the health outcomes and quality of life of preterm infants and their families.

## Materials and methods

This prospective interventional study was conducted in the postnatal ward of M S Ramaiah Hospital, Bengaluru, India, from November 2018 to September 2019. The study population consisted of stable preterm neonates with a corrected gestational age of 34 to 37 weeks and weighing between 1,500 and 2,000 grams at the time of enrollment. Preterm neonates with major congenital anomalies, chromosomal abnormalities, neurological disorders, those on intravenous fluids, inotropes, antibiotics, or other sick neonates with conditions such as sepsis or meningitis were excluded from the study.

The sample size was calculated based on a previous study that found a significant difference in temperature between the two bathing methods. Considering a mean temperature difference of 0.2°C between the two groups, a confidence level of 95% (alpha error 5%), and a power of 80% (beta error), the sample size was calculated to be 38 in each group using an appropriate formula.

Clearance from the Institutional Ethics Committee was obtained before the commencement of the study. Parents of the eligible preterm neonates were explained about the study objectives in detail, and written informed consent was obtained. The neonates were then allocated to two groups using a continuous sampling method: Group A (sponge bath followed by KMC) and Group B (immersion bath followed by KMC), with 38 neonates in each group.

In Group A, the preterm neonates received a sponge bath for two minutes using sterile gauze soaked in water at a temperature of 98.6-100.4°F (37-38°C). The bath was given by a trained nurse under the supervision of the investigator. The neonates were undressed, and the bathing pattern followed was trunk, genitalia, limbs, head, and neck. After the bath, the neonates were immediately dried with sterile gauze.

In Group B, the preterm neonates received an immersion bath for two minutes in a tub filled with water at a temperature of 98.6-100.4°F (37-38°C). The bath was given by a trained nurse under the supervision of the investigator. The neonates were undressed and immersed up to the neck, following the same bathing pattern as in Group A. After the bath, the neonates were immediately dried with sterile warm gauze.

In both groups, the axillary temperature was measured using a digital thermometer before the bath and at 15, 30, 45, and 60 minutes after the bath. Heart rate and oxygen saturation levels were also recorded using a stethoscope and a portable pulse oximeter, respectively. Immediately after the bath, the neonates in both groups received KMC for one hour. During KMC, the neonates were placed in an upright position, with direct skin-to-skin contact between the mother's chest and the neonate's chest and abdomen. The mother was seated comfortably in a reclining position, and the neonate was dressed only in a diaper and a cap.

The data collected were analyzed using appropriate statistical methods to compare the outcomes between the two groups and to determine the effectiveness of immersion bath followed by KMC and sponge bath followed by KMC on the thermal stability of preterm infants.

## Results

The study included a total of 76 preterm neonates, with 38 neonates in each group (sponge bath and immersion bath). The gender distribution across the two groups was similar, with a higher proportion of male neonates in both groups (Table 1). In the sponge bath group, there were 26 (68.4%) male and 12 (31.6%) female neonates, while in the immersion bath group, there were 23 (60.5%) male and 15 (39.5%) female neonates. Overall, 49 (64.5%) neonates were male, and 27 (35.5%) were female.

**Table 1:** Gender distribution in sponge bath and immersion bath groups

Group	Male (%)	Female (%)	Total
Sponge bath	26 (68.4)	12 (31.6	38
Immersion bath	23 (60.5)	15 (39.5)	38
Total	49 (64.5)	27 (35.5)	76

The weight distribution at enrollment was also comparable between the two groups (Table 2). In the sponge bath group, 29 (76.3%) neonates weighed between 1,500 and 1,750 grams, and 9 (23.7%) weighed between 1,751 and 2,000 grams. Similarly, in the immersion bath group, 25 (65.8%) neonates weighed between 1,500 and 1,750 grams, and 13 (34.2%) weighed between 1,751 and 2,000 grams. Overall, 54 (71.1%) neonates weighed between 1,500 and 1,750 grams, and 22 (28.9%) weighed between 1,751 and 2,000 grams.

**Table 2:** Weight at enrollment in sponge bath and immersion bath groups

Group	1,500-1,750 grams (%)	1,751-2,000 grams (%)	Total
Sponge bath	29 (76.3)	9 (23.7)	38
Immersion bath	25 (65.8)	13 (34.2)	38
Total	54 (71.1)	22 (28.9)	76

Table 3 compares the mean axillary temperature between the sponge bath and immersion bath groups when followed by KMC. The mean axillary temperature was significantly higher in the immersion bath group compared to the sponge bath group at all time points, except at 60 minutes post-bath. Before the bath, the mean axillary temperature was 97.6°F (SD=0.509) in the immersion bath group and 97.1°F (SD=1.192) in the sponge bath group, with a mean difference of -1.3°F (SE=0.205) and a t-value of 41.040 (p<0.001).

**Table 3:** Comparison of mean axillary temperature between sponge bath and immersion bath groups – KMC

Time interval	Sponge bath (n=38)	Immersion bath (n=38)	Mean diff	SE diff	t-value	P-value
	Mean (SD)	Mean (SD)				
Before	97.1 (1.192)	97.6 (0.509)	-1.3	0.205	41.04	<0.001
Immediate	96.3 (1.081)	97.6 (0.649)	-1.3	0.205	41.04	<0.001
15 min	96.8 (0.933)	97.6 (0.564)	-0.8	0.177	19.669	<0.001
30 min	97.3 (0.925)	97.9 (0.514)	-0.6	0.172	11.891	0.001
45 min	97.7 (0.802)	98.1 (0.429)	-0.5	0.147	9.973	0.002
60 min	98.0 (0.650)	98.2 (0.428)	-0.2	0.126	2.925	0.091

Immediately after the bath, the mean axillary temperature was 97.6°F (SD=0.649) in the immersion bath group and 96.3°F (SD=1.081) in the sponge bath group, with a mean difference of -1.3°F (SE=0.205) and a t-value of 41.040 (p<0.001). At 15 minutes post-bath, the mean axillary temperature was 97.6°F (SD=0.564) in the immersion bath group and 96.8°F (SD=0.933) in the sponge bath group, with a mean difference of -0.8°F (SE=0.177) and a t-value of 19.669 (p<0.001).

At 30 minutes post-bath, the mean axillary temperature was 97.9°F (SD=0.514) in the immersion bath group and 97.3°F (SD=0.925) in the sponge bath group, with a mean difference of -0.6°F (SE=0.172) and a t-value of 11.891 (p=0.001). At 45 minutes post-bath, the mean axillary temperature was 98.1°F (SD=0.429) in the immersion bath group and 97.7°F (SD=0.802) in the sponge bath group, with a mean difference of -0.5°F (SE=0.147) and a t-value of 9.973 (p=0.002).

At 60 minutes post-bath, the mean axillary temperature was 98.2°F (SD=0.428) in the immersion bath group and 98.0°F (SD=0.650) in the sponge bath group, with a mean difference of -0.2°F (SE=0.126) and a t-value of 2.925 (p=0.091). This difference was not statistically significant.

In summary, the study found that preterm neonates who received an immersion bath followed by KMC had significantly higher axillary temperatures compared to those who received a sponge bath followed by KMC at all time points post-bath, except at 60 minutes, indicating better thermal stability in the immersion bath group.

## Discussion

The present study compared the effects of immersion bath followed by KMC versus sponge bath followed by KMC on the thermal stability of preterm neonates. The results demonstrated that preterm neonates who received an immersion bath followed by KMC had significantly higher axillary temperatures than those who received a sponge bath followed by KMC at all time points post-bath, except at 60 minutes.

These findings are consistent with previous studies investigating the impact of bathing methods on the thermal stability of preterm neonates. In a randomized controlled trial by Edraki et al., preterm neonates who received an immersion bath had significantly higher axillary temperatures at 10 minutes (36.8°C vs. 36.5°C, p<0.001) and 30 minutes (36.8°C vs. 36.6°C, p=0.003) after the bath compared to those who received a sponge bath [8]. Similarly, a study by Loring et al. found that immersion bathing resulted in higher axillary temperatures (36.8°C vs. 36.4°C, p<0.001) and lower heat loss (18.6 kcal/kg/day vs. 21.5 kcal/kg/day, p<0.001) compared to sponge bathing in preterm neonates [6].

The better thermal stability observed in the immersion bath group in the present study can be attributed to several factors. Immersion bathing allows for a more uniform distribution of heat across the body surface, reducing evaporative heat loss [11]. Additionally, the water used in immersion bathing has a higher heat capacity than air, which helps maintain the neonate's body temperature [12].

The combination of immersion bathing and KMC may synergistically promote thermal stability in preterm neonates. KMC is an effective method for preventing hypothermia in preterm neonates [3]. A meta-analysis by Boundy et al. found that KMC was associated with a 36% reduction in the risk of hypothermia (relative risk (RR)=0.64; 95% confidence interval (CI), 0.46-0.89) compared to conventional care [4]. In the present study, both the sponge bath and immersion bath groups received KMC after bathing, which may have contributed to the overall improvement in thermal stability compared to pre-bath temperatures.

However, the immersion bath group demonstrated better thermal stability than the sponge bath group, even when both were followed by KMC. This finding suggests that the choice of bathing method may have an independent effect on thermal stability, regardless of the post-bath care provided. A study by Freitas et al. compared the effects of immersion bath followed by KMC versus sponge bath followed by radiant warmer care in preterm neonates [10]. They found that the immersion bath group had significantly higher axillary (36.5°C vs. 36.2°C, p=0.026) and abdominal (36.6°C vs. 36.3°C, p=0.001) temperatures compared to the sponge bath group at 30 minutes after bathing.

The present study did not find a significant difference in axillary temperature between the immersion bath and sponge bath groups at 60 minutes post-bath. This may be due to the prolonged effect of KMC in both groups, which helped maintain thermal stability over time. A study by Srivastava et al. found that KMC was effective in maintaining axillary temperature within the normal range (36.5°C-37.5°C) for up to six hours after the intervention [13].

The findings of this study have important implications for clinical practice. Immersion bathing followed by KMC appears to be a superior method for maintaining thermal stability in preterm neonates compared sponge bathing followed by KMC. Healthcare providers should consider adopting this approach as the preferred bathing method for preterm neonates, especially in settings where resources are limited and access to advanced warming equipment may be scarce.

However, the study has some limitations that should be acknowledged. The sample size was relatively small, and the study was conducted at a single center, which may limit the generalizability of the findings. Additionally, the study did not assess other important outcomes, such as skin integrity, infection rates, or maternal satisfaction, which could be affected by the bathing method.

This study provides evidence that immersion bathing followed by KMC is more effective than sponge bathing followed by KMC in maintaining thermal stability in preterm neonates. Further research is needed to confirm these findings in larger, multi-center studies and to evaluate the long-term effects of different bathing methods on various health outcomes in preterm neonates.

## Conclusions

In this prospective interventional study, we compared the effects of immersion bath followed by KMC versus sponge bath followed by KMC on the thermal stability of preterm neonates. The results demonstrated that preterm neonates who received an immersion bath followed by KMC had significantly higher axillary temperatures than those who received a sponge bath followed by KMC at all time points post-bath, except at 60 minutes. The findings of this study have important implications for clinical practice, suggesting that immersion bathing followed by KMC should be considered the preferred method for bathing preterm neonates to promote better thermal stability. Healthcare providers should adopt this approach, especially in resource-limited settings where access to advanced warming equipment may be limited.

In conclusion, this study provides evidence that immersion bathing followed by KMC is a superior method for maintaining thermal stability in preterm neonates compared to sponge bathing followed by KMC. The implementation of this approach in clinical practice has the potential to improve health outcomes and quality of life for preterm neonates and their families.
